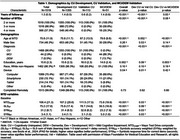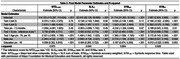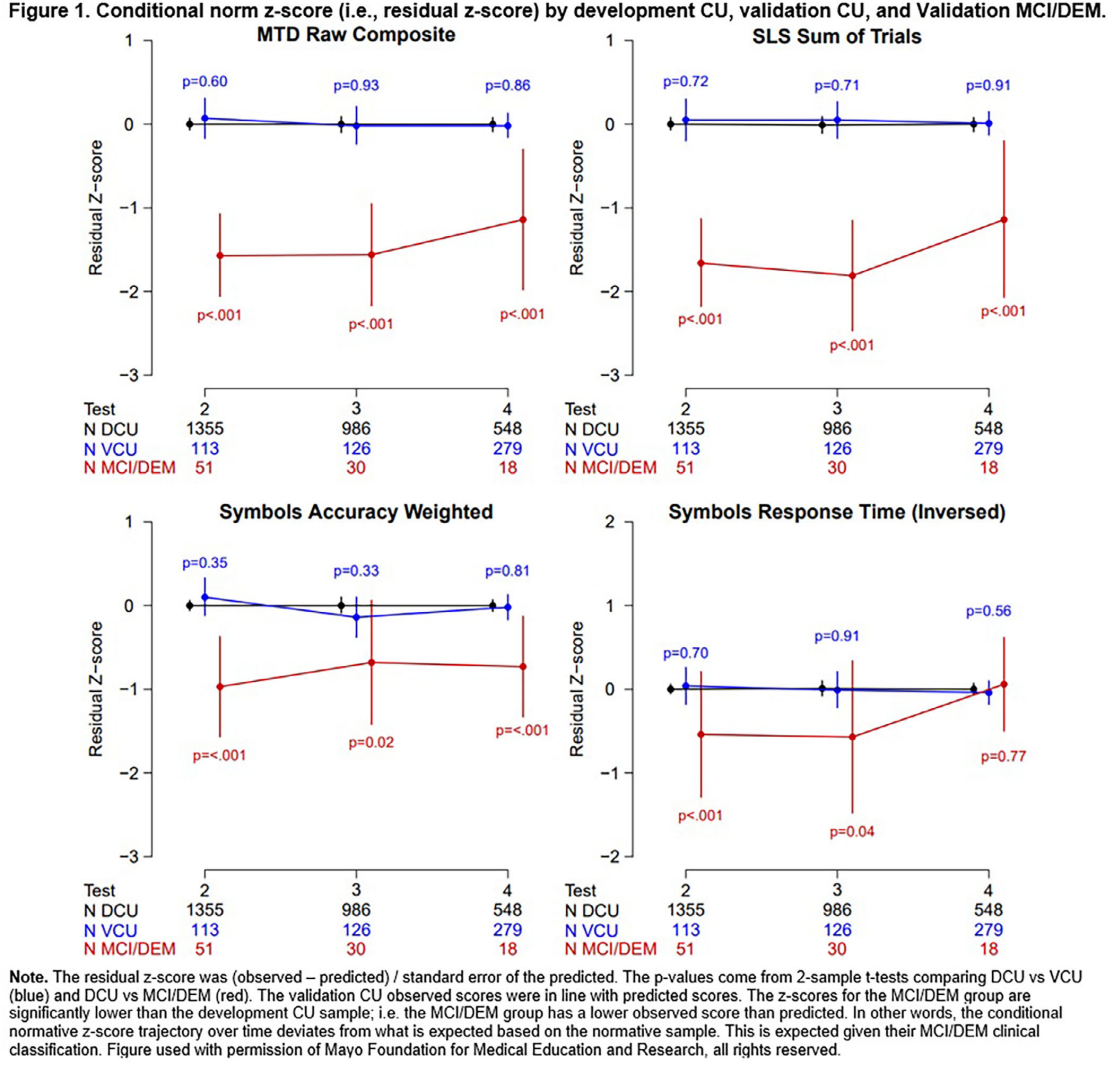# Mayo Normative Studies: a conditional normative model for longitudinal change in performance on remote digital cognitive measures including the Stricker Learning Span, Symbols Test and Mayo Test Drive Screening Battery Composite

**DOI:** 10.1002/alz70857_099872

**Published:** 2025-12-24

**Authors:** Ryan D. Frank, Teresa J. Christianson, Winnie Z Fan, Morgan A Hughes, Walter K. Kremers, John L. Stricker, Mary M. Machulda, John A. Lucas, Jason J. Hassenstab, Paula Aduen, Gregory S Day, Neill R. Graff‐Radford, Clifford R. Jack, Jonathan Graff‐Radford, Ronald Petersen, Nikki H. Stricker

**Affiliations:** ^1^ Mayo Clinic, Rochester, MN, USA; ^2^ Department of Information Technology, Mayo Clinic, Rochester, MN, USA; ^3^ Department of Psychiatry and Psychology, Mayo Clinic, Rochester, MN, USA; ^4^ Mayo Clinic in Florida, Jacksonville, FL, USA; ^5^ Washington University in St. Louis, St. Louis, MO, USA; ^6^ Department of Neurology, Mayo Clinic, Rochester, MN, USA

## Abstract

**Background:**

Self‐administered and remote digital cognitive assessments provide a scalable method for screening and longitudinal cognitive monitoring. Mayo Test Drive (MTD) is one such assessment with previously demonstrated usability, reliability, validity and multi‐device compatibility. MTD includes a computer‐adaptive word list memory test (Stricker Learning Span) and a measure of processing speed/executive functioning (Symbols Test) that combine to provide an MTD Composite. This study aimed to develop a conditional normative model predicting longitudinal cognitive change for MTD, and to validate the model in two independent samples of cognitively unimpaired (CU) and mild cognitive impairment (MCI)/dementia (DEM) participants.

**Method:**

1355 CU participants at initial MTD assessment with 2‐4 consecutive MTD assessments every 7.5 months comprised the development dataset. Linear mixed effects models were used to construct a conditional normative model for MTD assessments 2‐4. The baseline model was chosen *a priori*, consisting of age, sex, education, baseline score, number of assessments, and time. Device type, race, and baseline score by time interactions were also considered. Variables were only added if a >1% increment in r‐squared was observed. The models were externally validated in 113 CU and 51 MCI/DEM patients. Following model development, the data were updated and additional follow‐up MTD assessments were added for many participants, including the addition of additional CU validation data at test 3 (*N* = 126) and 4 (*N* = 279). T‐tests compared observed and predicted differences and residual z‐scores between development and validation at each follow‐up assessment.

**Result:**

Table 1 presents demographic variables. No additional variables increased the r‐squared by >1%. The final models are presented in Table 2. Age, baseline score, sex, and education were significant. Residual z‐scores were not significantly different between CU development and CU validation cohorts, indicating observed scores were in line with model predicted scores. Residual z‐scores were significantly worse among MCI/DEM compared to CU development in 11/12 comparisons (Figure 1), indicating observed scores were below model predicted expectations and implying sensitivity to longitudinal cognitive decline.

**Conclusion:**

These longitudinal normative models can be used to identify individuals with follow‐up scores outside of expected predicted ranges, signifying abnormal longitudinal cognitive decline.